# Omicron-Induced Immune Thrombocytopenia: A Case Report

**DOI:** 10.7759/cureus.39648

**Published:** 2023-05-29

**Authors:** Haneen A Toba, Mohammad Abu-Tineh, Awni Alshurafa, Khalid Ahmed, Baian Mohammed, Mahmoud M Altayyan, Mohammed Abdulgayoom, Mohamed A Yassin

**Affiliations:** 1 Department of Internal Medicine, Hamad Medical Corporation, Doha, QAT; 2 Department of Medical Oncology, Hematology and Bone Marrow Transplant Section, National Center for Cancer Care and Research, Hamad Medical Corporation, Doha, QAT; 3 Department of Internal Medicine, Hamad General Hospital, Doha, QAT; 4 Department of Hematology and Oncology, Hamad General Hospital, Doha, QAT

**Keywords:** covid-19, steroids, thrombocytopenia, ivig, itp

## Abstract

Coronavirus disease 2019 is a systemic infection that significantly impacts the hematopoietic system and hemostasis. Among the hematological manifestations described, severe and symptomatic thrombocytopenia is rare. Immune thrombocytopenia (ITP), also known as idiopathic thrombocytopenic purpura or immune thrombocytopenic purpura, is an acquired thrombocytopenia caused by autoantibodies against platelet antigens. It is one of the more common causes of thrombocytopenia in otherwise asymptomatic adults. Here, we report the case of a patient who developed ITP after a severe acute respiratory syndrome coronavirus 2 infection to highlight the rarer hematological manifestations of the disease and the changes in treatment.

## Introduction

Severe acute respiratory syndrome coronavirus 2 (SARS-CoV-2) was originally discovered as the cause of a wave of pneumonia cases in Wuhan, Hubei Province, China, near the end of 2019. It soon spread throughout the world, causing a global epidemic [1-3]. Many risk factors may aggravate the condition, such as diabetes, hypertension, and eosinophilia [4-6].

Immune thrombocytopenic purpura (ITP) is a hematologic disorder in which antibodies coating platelets cause platelet destruction in the spleen, resulting in a low platelet count and an increased tendency to bleed [7]. Cases of ITP have been recorded in coronavirus disease 2019 (COVID-19) vaccination recipients with a platelet count of lower than 100,000, including new-onset ITP and worsening of pre-existing ITP [8]. However, there is no evidence to suggest a higher rate of ITP in individuals who receive any of the COVID-19 vaccines compared to the background rate in the population.

## Case presentation

A previously healthy 41-year-old Nepalese male patient with a normal baseline platelet count presented with complaints of pinkish discoloration of urine, reddish spots on the skin, cough, and throat pain of two days duration. On examination, he was found to have a fever of 38.1°C and a diffuse petechial rash. No lymphadenopathy or organomegaly was noted. All other examinations were unremarkable. Labs showed severe thrombocytopenia, normocytic, normochromic anemia, and high C-reactive protein (Table 1). He tested positive for COVID-19 with a CT value of 17.68. It is worth mentioning that the patient had received two doses of the SARS-CoV-2 (Pfizer) vaccine. The last dose was one year before the infection.

**Table 1:** Laboratory values on admission.

Group	Detail	Value	Flags	Normal range
General hematology	White blood cell count	5.3 × 10^3^/µL	Normal	4.0–10.0
	Hemoglobin	11.3 g/dL	Low	13.0–17.0
	Mean corpuscular volume	86.4 fL	Normal	83.0–101.0
	Mean corpuscular hemoglobin	30.2 pg	Normal	27.0–32.0
	Platelet count	1 × 10^3^/µL	Critical	150–410
	*Reticulocyte* count	2.9%	High	0.5–2.5
Coagulation	International normalized ratio	1.3	NA	
	D-dimer	4.34 mg/L FEU	High	0.00–0.49
	Fibrinogen	4.30 g/L	High	2.00–4.10
Blood chemistry	C-reactive protein	60.4 mg/L	High	0.0–5.0
	Iron	16 µmol/L	Normal	6–35
	Total iron-binding capacity	50 µmol/L	Normal	45–80
	Transferrin	2.0 g/L	Normal	2.0–3.6
	Iron saturation	32%	Normal	15–45

**Figure 1:**
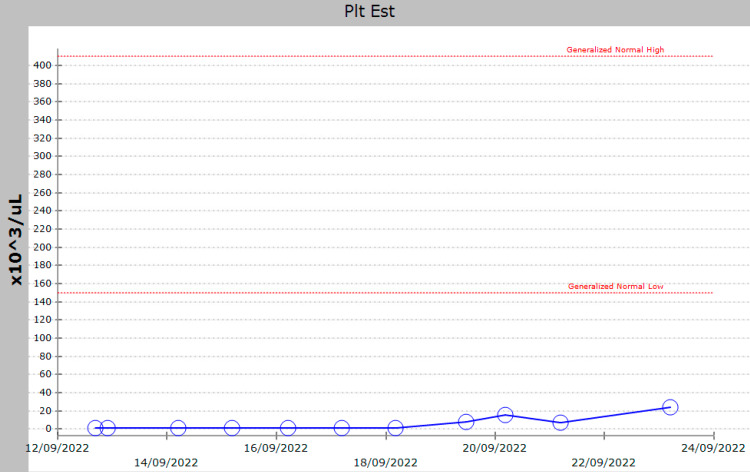
Platelet trend during admission.

His chest X-ray was normal, with no pneumonic consolidation. Peripheral smear showed bi-cytopenia (mild anemia with mild reticulocytosis) and marked thrombocytopenia with a picture suggestive of reactive changes to infection, without any evidence of hemolysis. Autoimmune workup (antinuclear antibodies), anti-cardiolipin, anti-B2 glycoprotein, complements, and direct antiglobulin test all returned negative. The impression was COVID-induced ITP, and the patient was started on intravenous immunoglobulin (IVIG) 1 g/kg for two days, followed by a dexamethasone pulse (20 mg for two days, followed by 40 mg for four days). The platelet counts gradually improved, reaching 24,000/µL on day 10 of admission (Figure 1).

## Discussion

Although the mechanism of thrombocytopenia in COVID-19 patients is not well understood yet, there are three potential mechanisms to consider. First, bone marrow cells are directly infected by a virus, which also prevents the production of platelets. Cytokine storm after a viral infection kills bone marrow progenitor cells and reduces platelet synthesis. Second, platelet aggregation in the lungs, which produces microthrombi. Lastly, immune system-mediated platelet destruction is caused by molecular mimicry between platelet membrane components (particularly glycoprotein) and viral antigens [9].

In one case report, a patient with ITP and COVID-19 experienced a subarachnoid hemorrhage. On admission, the patient was initiated on IVIG; the bleeding started on day 9. In another case report, a patient similar to ours with ITP associated with COVID-19 died after suffering an intracerebral hemorrhage [10].

ITP therapy does not aim to normalize the platelet count but to provide a safe platelet count to prevent clinically significant bleeding [11]. We used IVIG and glucocorticoids together because each has a unique mode of action and can be additive, although no direct trials have been recorded. Splenectomy, rituximab [12], or a thrombopoietin receptor agonist (TPO-RA) [13] are the three top choices for second-line treatment. All three are effective in raising the platelet count in most individuals.

A TPO-RA is considered a good choice for an individual who is especially concerned about immunosuppression following splenectomy or rituximab and who is less concerned about taking a medication for an extended period, including the associated costs and burdens. Temporary use of a TPO-RA may be appropriate during the COVID-19 pandemic to avoid immunosuppressive therapy.

## Conclusions

ITP post-SARS-CoV-2 infection has been reported in a few cases where there was a significant drop in platelet count. Based on the temporal profile and the exclusion of other etiologies, this case shows a possible association between COVID-19 and ITP.
